# Primum non nocere: earlier cessation of glucose monitoring is possible

**DOI:** 10.1007/s00431-018-3169-z

**Published:** 2018-05-30

**Authors:** Celine Blank, Jeroen van Dillen, Marije Hogeveen

**Affiliations:** 1 Department of Obstetrics and Gynaecology, Catharina Hospital, Michelangelolaan 2, 5623 EJ Eindhoven, the Netherlands; 2 Department of Electrical Engineering (Signal Processing Systems), Eindhoven Technical University, Flux, Groene loper 19, Postbus 513, 5600 MB Eindhoven, the Netherlands; 3 Department of Obstetrics and Gynaecology, Radboudumc, Geert Grooteplein Zuid 10, 6525 GA Nijmegen, the Netherlands; 4 Division of Neonatology, Department of Paediatrics, Radboudumc, Amalia Children’s Hospital, Geert Grooteplein Zuid 10, 6525 GA Nijmegen, the Netherlands

**Keywords:** Glucose monitoring, Neonatal hypoglycaemia, Newborns, Screening

## Abstract

Newborns are at relatively high risk for developing hypoglycaemia in the first 24 h after birth. Well-known risk factors are prematurity, small for gestational age (SGA) or large for gestational age (LGA), and maternal pre-existent or gestational diabetes mellitus. Prolonged hypoglycaemia is associated with poor neurodevelopmental outcomes; hence, prevention through proper monitoring and treatment is important. Given the ongoing debate concerning frequency and duration of screening for neonatal hypoglycaemia, therefore, we investigated the frequency and duration of glucose monitoring safe to discover neonatal hypoglycaemia in different risk groups. Data of newborns at risk for hypoglycaemia were retrospectively collected and analysed. Blood glucose concentrations were measured 1, 3, 6, 12, and 24 h after birth. Moderate hypoglycaemia was defined as a blood glucose concentration of < 2.2 mM and severe hypoglycaemia as a concentration of < 1.5 mM. Of 1570 newborns, 762 (48.5%) had at least one episode of hypoglycaemia in the first 24 h after birth; 30.6% of them had severe hypoglycaemia (all in the first 9 h after birth). Only three SGA and two LGA newborns had a first moderate asymptomatic hypoglycaemic episode beyond 12 h after birth. The incidence of hypoglycaemia increased with accumulation of multiple risk factors.

*Conclusion*: Safety of limiting the monitoring to 12 h still has to be carefully evaluated in the presence of SGA or LGA newborns; however, our results suggest that 12 h is enough for late preterm newborns (> 34 weeks) and maternal diabetes.

**Table:** 

**What is Known:** • *Newborns are at relatively high risk for developing hypoglycaemia and such hypoglycaemia is associated with adverse neurodevelopmental outcomes.* • *Proper glucose monitoring and prompt treatment in case of neonatal hypoglycaemia are necessary.*	
**What is New:** • *Glucose monitoring 12 h after birth is proficient for most newborns at risk.* • *Maternal diabetes leads to the highest risk of early neonatal hypoglycaemia and newborns with more than one risk factor are at increased risk of hypoglycaemia.*

## Introduction

To date, there is still little evidence and consensus regarding definition, indication, and frequency of screening for neonatal hypoglycaemia, one of the most frequently encountered problems in neonatology. Newborns are at relatively high risk for developing hypoglycaemia in the first 24 h after birth [10]. The incidence of hypoglycaemia in apparently healthy newborns varies widely (between 4 and 40%) depending amongst others on definition of hypoglycaemia, timing of glucose monitoring after delivery, and population studied [16, 21].

Risk factors for neonatal hypoglycaemia are prematurity (< 37 weeks of gestation), being small for gestational age (SGA) (birth weight ≤ 10th percentile for GA), large for gestational age (LGA) (birth weight ≥ 90th percentile for GA), and pre-existent maternal diabetes (diabetes mellitus type 1, type 2 (T1DM, T2DM)) or gestational diabetes mellitus (GDM) [2, 3, 11, 26].

In apparently healthy infants, glucose concentrations tend to decrease after birth, reach a nadir 1 h after birth, increase again, and stabilise after one day [2, 16]. The majority of first hypoglycaemic episodes occur in the first 24 h after birth (81%), from which 48% within 6 h [12].

Recurrent severe hypoglycaemia is associated with poor neurodevelopmental outcomes; however, duration and severity of hypoglycaemia to cause injury are unknown. The management of the asymptomatic infant at risk for low glucose remains controversial. Despite this, prevention of (prolonged) hypoglycaemia by screening and treatment seems rational [3, 13, 19].

Existing guidelines for screening and treatment recommend glucose monitoring in newborns at risk but vary in (1) definition of hypoglycaemia (between 2.2 and 2.6 mM), (2) total monitoring time (12–48 h after birth), and (3) time intervals (between 1 and 8 h). This is illustrating the lack of consensus regarding the frequency and duration of glucose monitoring deemed necessary and safe to discover neonatal hypoglycaemia [2, 7, 12, 15, 18]. Furthermore, different risk factors may prompt a different strategy. Therefore, we studied the onset of the first hypoglycaemic episode in newborns at risk according to their risk factors.

## Materials and methods

This is a retrospective observational cohort study. We enrolled all eligible newborns born and admitted at Radboudumc Amalia Children’s Hospital Nijmegen, the Netherlands, from a four-year period (2010–2013). Inclusion criteria were gestational age (GA) ≥ 34 weeks and screening indication according to our local guideline: prematurity (< 37 weeks of gestation), SGA, LGA, and maternal diabetes of any kind (identical to recommendations by the American Academy of Pediatrics (AAP)) [3, 4]. Exclusion criteria were severe asphyxia (Apgar 5 min after birth ≤ 3) [1] and death within the first 24 h after birth. In the first 24 h after birth, moderate hypoglycaemia and severe hypoglycaemia were defined as a blood glucose concentration (GC) between 1.5–2.1 mM and below 1.5 mM, respectively, moderate and severe reflecting therapy in this case. Blood glucose concentrations were measured on capillary blood samples taken by several point-of-care (POC) glucose meters during this period (HemoCue Glu201DM, Nova StatStrip GluCard memory PC, Roche OMNI-56 blood gas analyser and Siemens Rapid lab 1265). In case of hypoglycaemia (GC < 2.2 mM), capillary blood was sent to the laboratory for confirmation.

Blood glucose samples were taken 1, 3, 6, 12, and 24 h after birth according to local guidelines [9]. In case of any clinical signs of hypoglycaemia, such as twitching and drowsiness, additional glucose samples were taken.

Feeding was initiated in the first hour after birth. Breastfeeding was encouraged throughout pregnancy, but the ultimate choice between breastfeeding and formula feeding was made by the parents. In our hospital (as in the Netherlands), approximately 80% of women start with exclusive breastfeeding post-delivery [22]. Feeding is continued at least every 3 h, with breastfeeding sometimes more often. Newborns with moderate hypoglycaemia were treated with increased enteral carbohydrate intake, either through more frequent breast feedings or (additional) formula feeding. In case of repeated moderate hypoglycaemia (≥ 3×), continuous intravenous glucose 10% was started after administration of 2 ml/kg bolus. Newborns with severe hypoglycaemia were immediately treated with continuous intravenous glucose 10% and a bolus.

Birth percentiles, SGA (< 10th percentile) or LGA (> 90th percentile), were calculated using Dutch birth weight curves (https://www.perined.nl/producten/geboortegewichtcurven).

Relevant maternal and neonatal information and GC were extracted from patient files. Our primary outcome was time of first hypoglycaemia, which we studied in all risk factors separately. When hypoglycaemia was described in discharge correspondence but data on timing of blood glucose concentrations were missing of more than two standardised sample moments, cases were only used for overall analysis.

For statistical analysis, SPSS software version 20 was used. Normally, distributed data were expressed as percentage and mean (SD). Median (range) was used for skewed distributed data. For 2 × 2 tables, chi-square test was performed. Multivariate logistic regression analyses were used to examine the association between (severe) hypoglycaemia and possible risk factors, including screening indications, background information, and other complications. A *p* value < 0.05 was considered statistically significant.

This study was exempt from Regional Ethics Review Board approval, under the legal requirements for clinical research in the Netherlands.

## Results

**Fig. 1:**
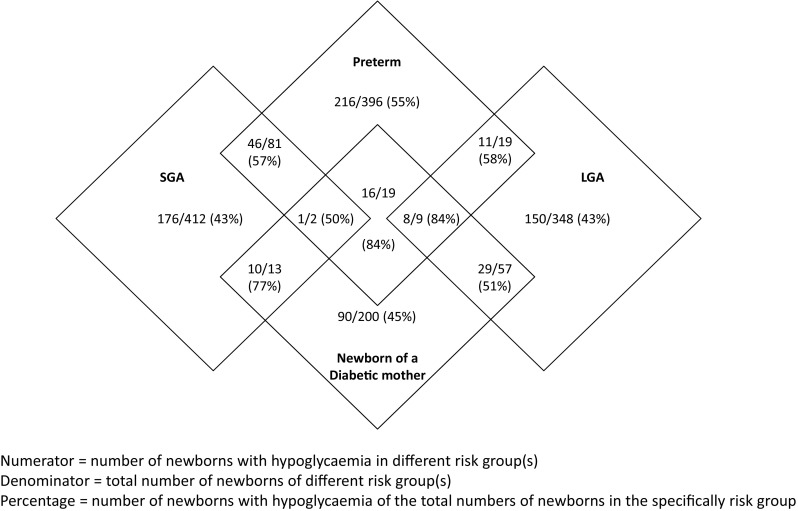
Neonatal hypoglycaemia with different combinations of risk factors

**Fig. 2:**
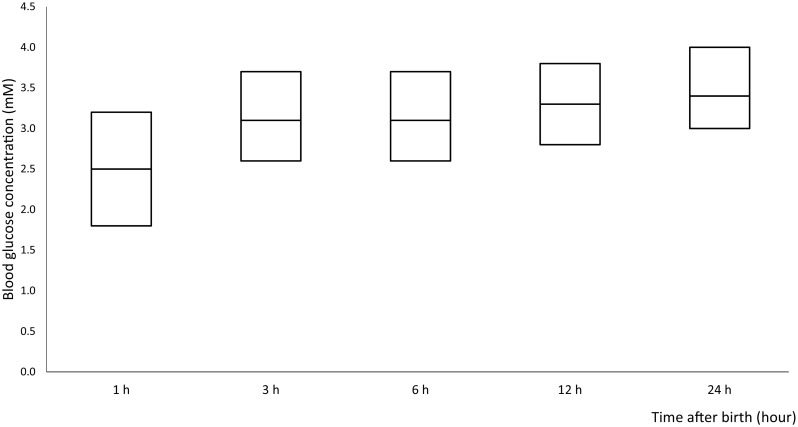
Median blood glucose concentration of total newborns (median, 25th and 75th percentile)

We enrolled 1628 newborns with risk factors for hypoglycaemia of which 1573 met our inclusion and exclusion criteria. Data of three newborns were unavailable. The final analysis was performed on data from 1570 newborns. In 327 cases, data of more than two sample moments were missing. These cases were only used for the overall analysis (hypoglycaemia 125/327). Newborns at risk were premature (33.5%), SGA (32.4%), LGA (27.6%), or born to a diabetic mother (19.1%) (Table 1). Most newborns had one risk factor (87.1%), 12.1% two risk factors, and 0.7% three risk factors (Fig. 1). In the first hour after birth, GCs for this cohort reach their lowest values. After that, blood GCs remain stable at a value of 3.1–3.4 mM (Fig. 2).

**Table 1:** Maternal, gestational, and newborn characteristics

	Overall	Glucose < 2.2 mM ‘hypoglycaemia’	Glucose < 1.5 mM ‘severe hypoglycaemia’
Total number of newborns	1570	762/1570 (48.5%)	271/1570 (17.2%)
Gestational age, weeks*	38.29 (36.43–39.86)	37.86 (36.29–39.43)	37.49 (36.14–38.86)
Birth weight, g**	3106 ± 814	3043 ± 819	2980 ± 783
Sex			
Male	804/1570 (51.2%)	395/804 (49.1%)	133/804 (16.5%)
Females	766/1570 (48.8%)	367/766 (47.9%)	138/766 (18.0%)
Single/multiple birth			
Singleton	1385/1570 (88%)	657/1385 (47.4%)	238/1385 (17.2%)
Twin	164/1570 (10.4%)	72/165 (43.9%)	27/165 (16.4%)
Triplet	20/1570 (1.3%)	8/20 (40.0%)	6/20 (30.0%)
Preterm delivery			
Total preterm deliveries	526/1570 (33.5%)	298/526 (56.7%)	115/526 (21.9%)
34–35 weeks	102/526 (19.4%)	51/102 (50.0%)	22/102 (21.6%)
35–36 weeks	163/526 (31.0%)	85/163 (52.1%)	36/163 (22.1%)
36–37 weeks	261/526 (49.6%)	162/261 (62.1%)	57/261 (21.8%)
Weight			
LGA (*p* > 90)	433/1570 (27.6%)	198/433 (45.7%)	61/433 (14.1%)
Extremely LGA (*p* > 97.7)	114/1570 (7.4%)	52/114 (45.6%)	17/114 (14.9%)
SGA (*p* < 10)	508/1570 (32.4%)	233/508 (45.9%)	115/508 (22.6%)
Extremely SGA (*p* < 2.3)	131/1570 (8.3%)	69/131 (52.7%)	22/131 (16.8%)
Diabetes			
Total	300/1570 (19.1%)	154/300 (51.3%)	66/300 (22.0%)
T1DM/ T2DM	41/300 (13.7%)	33/41 (80.5%)	18/41 (43.9%)
GDM	259/300 (86.3%)	121/259 (46.7%)	48/259 (18.5%)

Data are number (percentage). Percentages are of total number of newborns or of individual and overall numbers of newborns in the different groups

*Median (25th–75th percentile)

**Mean (SD)

Of 1570 patients at risk, 762 (48.5%) suffered from at least one episode of hypoglycaemia. One-third of hypoglycaemic episodes were severe (< 1.5 mM), with maternal pre-existing diabetes as the main risk factor (54.5%) (Table 1). The prevalence of hypoglycaemia is similar in different birth weight groups (*p* > 0.05) (Table 1). Pre-existent maternal diabetes led to early hypoglycaemia (first hour after birth) in 21 cases (75%) (Table 2).

**Table 2:** Timing of first hypoglycaemia according to screening indication

Timing of first hypoglycaemia	All newborns	T1DM, T2DM	GDM	Preterm	SGA	LGA
	*N* = 1570*	*N* = 41	*N* = 259	*N* = 526	*N* = 508	*N* = 433
0–1 h after birth	512/1428	21/34	83/237	207/457	135/454	134/386
> 1–2 h after birth	19/60	0/2	5/11	8/27	3/16	6/16
> 2–3 h after birth	102/888	5/13	12/149	33/238	45/317	16/240
> 3–5 h after birth	3/22	0/0	1/5	1/7	0/6	1/6
> 5–9 h after birth	71/797	1/9	8/136	14/210	32/279	24/224
> 9–12 h after birth	19/768	1/8	4/135	7/206	3/257	4/218
> 12–15 h after birth	1/33	0/0	0/4	0/11	0/11	1/10
> 15–18 h after birth	0/65	0/1	0/9	0/26	0/17	0/17
> 18–24 h after birth	4/667	0/7	0/119	0/177	3/223	1/191

*There are newborns with more than one risk factor

In GDM, the majority of newborns (100/113; 88.5%) had a hypoglycaemia within 3 h after birth. Hypoglycaemia occurring more than 12 h after birth for the first time was seen in only five newborns (Table 2). One SGA newborn had a GC of 2.1 mM 15 h after birth, and three LGA newborns and one SGA newborn had moderate hypoglycaemias (GC 1.7, 1.9, 2.1, and 2.1 mM respectively) 24 h after birth. These newborns were all treated with additional feedings only.

In the group with other complications, e.g. congenital abnormalities, respiratory distress, or (risk for) infection, significantly less newborns developed a hypoglycaemic episode (*p* = 0.04).

The overall prevalence of hypoglycaemia increased with increasing number of risk factors (one risk factor (43–55%), comparing to the group with two risk factors (51–84%, *p* = 0.011) and the group with three risk factors (50–89%, *p* = 0.004)) (Fig. 1).

Multivariate logistic regression analysis including all potential risk factors, sex, single/multiple birth, and other complications (congenital abnormalities, respiratory distress and (risk for) infection) showed that hypoglycaemia was significant associated with prematurity (OR = 1.545, 95% CI 1.00–2.02, *p* < 0.05), pre-existent DM (T1DM and T2DM combined) (OR = 4.23, 95% CI 1.81–10.32, *p* = 0.001), and other complications (OR = 0.70, 95% CI 0.53–0.94, *p* = 0.018). Also, in case of severe hypoglycaemia, significant association was found with newborns born to mothers with pre-existent DM (OR = 4.74, 95% CI 2.44–9.20, *p* < 0.001) and prematurity (OR = 1.84, 95% CI 1.24–2.73, *p* = 0.002).

## Discussion

The overall incidence of hypoglycaemia in our cohort of neonates at risk was 48.5%. For most newborns at risk, it is safe to stop monitoring GCs 12 h after birth. The highest incidence of hypoglycaemia was related to maternal diabetes and there is an increasing risk of hypoglycaemia with increasing number of risk factors. The majority of first hypoglycaemias occurred within 3 h after birth, comparable to other studies [14, 16].

The overall incidence of hypoglycaemia in our cohort is rather high; other studies report 6.8–51% [6, 12, 20, 23]. This might be due to our selection criteria for newborns at risk for hypoglycaemia although our screening indications are identical to those recommended by the AAP [3]. Other possible explanations are our standardised and frequent monitoring and difference in GC cut-off point to define hypoglycaemia. Hypoglycaemia was defined as a GC < 2.6 mM by Harris et al. [12] (overall incidence 51%) and a GC < 1.6 mM by Schaefer-Graf et al. [23] (overall incidence 25.2%); by means of this, incidence of hypoglycaemia might be influenced. Our lowest level of action is 2.2 mM in the first 24 h which is comparable to the actionable range recommended by the AAP (4–24 h after birth, 1.9–2.5 mM) [3].

A wide range of policies regarding duration of glucose concentration monitoring exists [2, 7, 12, 15]. Some authors state that the same screening strategy should be applied to each risk group while others suggest to differentiate between risk groups [12]. Different studies suggest that LGA newborns and newborns of diabetic mothers should have their monitoring discontinued 12 h after birth (if GC is ≥ 2.6 mM) [2, 7] and SGA newborns and late preterm newborns after 24 h [2], 36 h [7], or 48 h [15].

The high incidence of hypoglycaemia in offspring of mothers with pre-existent DM (81%) was comparable to the incidence seen in the study of Maayan Metzger et al. (54% GC < 2.6 mM and 83% GC < 2.2 mM) [18] but rather high compared to the study of Harris et al. (49%) [12]. However, in the latter study, no distinction between maternal pre-existent DM and GDM was defined.

More than one risk factor resulted in higher incidence (Fig. 2) which is in contrast to the study of Harris et al. in which no significant difference was found [12]. This might be due to the relative small sample size in their study.

The nadir 1–2 h after birth in blood GC is also observed in apparently health newborns and is considered part of normal adaptation to postnatal life by several authors, although it is not consistently described [2, 14, 16].

Our data illustrate that 96.7% (710/734) of all newborns who developed hypoglycaemia had the first hypoglycaemia within 6 h after birth. Since there were only five cases of mild asymptomatic hypoglycaemia beyond 12 h, all treated with additional feeding only, one could argue to stop glucose monitoring after 12 h for all risk groups. Safety of limiting the monitoring to 12 h still has to be carefully evaluated in the presence of SGA or LGA newborns.

Some limitations of this study should be addressed. Due to the retrospective design, it was not possible to obtain the exact glucose concentrations in 327 cases. Since the introduction of the electronic patient files, values of blood glucose concentrations are directly stored in these files. Furthermore, not all samples were obtained at the exact prescribed time points, which was mainly due to breastfeeding on demand and sampling just prior to intake (recommended clinical practice). Also, in this time period, several blood glucose meters and analysers were used. It is known that results of POC glucose meters in the critical range may be unreliable [24]. To minimise misdiagnosis, blood samples, which showed a GC below 2.2 mM, were retested in the clinical laboratory. Moreover, variance in reliability of POC glucose meters is depending on variability in instrument analytical performance [17] and interaction between users and POC glucose meters [8]. Continuous glucose monitoring (CGM) has been studied in neonates and results are promising. CGM could potentially decrease number of blood samples and the exposure to hypoglycaemia. Furthermore, it is feasible, calibration can be as low as 12 hourly, and it has limited side effects even in premature newborns (birth weight < 1500 g) and is well tolerated [5]. However, no association with improved clinical outcomes has been confirmed yet. There are technical issues to be improved as well, for example the provided range of 2.2 to 22.0 mM, which is insufficient to detect neonatal hypoglycaemia [25]. Randomised trials should demonstrate long-term outcome data using CGM [5, 25]. In our centre, ~ 85% is Caucasian; therefore, our results may need to be confirmed in other ethnic populations.

To our knowledge, this is the largest published study that specifically investigated the length of time that is necessary in neonatal glucose monitoring. The newborns were selected based on strict inclusion criteria for each separate risk factor and GC were measured using a standardised protocol.

In conclusion, the onset of first hypoglycaemia did not occur beyond 12 h of glucose monitoring after birth in prematurity and maternal diabetes in the observed time frame. Multiple risk factors and an inappropriate birth weight for gestational age may increase the risk of hypoglycaemic episode.

## Abbreviations

CGM: Continuous glucose monitoring
DM: Diabetes mellitus
GC: Glucose concentration
GDM: Gestational diabetes mellitus
LGA: Large for gestational age
mM: Millimolar (1 mmol/litre)
POC: Point-of-care
SD: Standard deviation
SGA: Large for gestational age
T1DM: Diabetes mellitus type 1
T2DM: Diabetes mellitus type 2
